# Complete Chloroplast Genome Sequences of Three *Canna* Species: Genome Characterization, Comparative Analyses, and Phylogenetic Relationships Within Zingiberales

**DOI:** 10.3390/cimb47040222

**Published:** 2025-03-25

**Authors:** Linhe Sun, Jixiang Liu, Fangyu Liu, Wei Wang, Yajun Chang, Dongrui Yao

**Affiliations:** 1 Jiangsu Key Laboratory for the Research and Utilization of Plant Resources, Institute of Botany, Jiangsu Province and Chinese Academy of Sciences (Nanjing Botanical Garden Mem. Sun Yat-Sen), Nanjing 210014, China; linhesun@cnbg.net (L.S.); ljx891654338@163.com (J.L.); shuishengzu@126.com (D.Y.); 2 Jiangsu Engineering Research Center of Aquatic Plant Resources and Water Environment Remediation, Nanjing 210014, China; 2021104111@stu.njau.edu.cn (F.L.); izjwang@163.com (W.W.); 3 College of Horticulture, Nanjing Agricultural University, Nanjing 210095, China; 4 College of Biology and the Environment, Nanjing Forestry University, Nanjing 210037, China

**Keywords:** chloroplast genome, *Canna*, Zingiberales, genome structure, phylogeny

## Abstract

*Canna*, the sole member of the Cannaceae family, is widely cultivated as an ornamental plant for its decorative flowers and foliage and is also a potential tuber crop due to its high starch content. This study sequenced, assembled, and analyzed the complete chloroplast (cp) genomes of three common *Canna* species with distinct leaf colors (green, purple, and variegated). The four cp genomes ranged from 164,427 to 164,509 bp in length, had a GC content of 36.23–36.25%, and exhibited identical gene content and codon preferences. Each genome contained 130 genes, including 110 unique genes (78 protein-coding genes, four of unknown function, four rRNAs, and 28 tRNAs), 18 duplicated genes located in the IR regions (six protein-coding genes, two of unknown function, four rRNAs, and eight tRNAs), and two *trnM-CAU* genes in the LSC region. SSR and long-repeat showed differences in long repeats numbers and distributions among the four cp genomes, highlighting potential molecular markers for *Canna* species identification and breeding. Comparative analysis showed high conservation across *Canna* cp genomes. Phylogenetic analysis confirmed a close relationship between Cannaceae and Marantaceae and supported a [Musaeceae (Cannaceae + Marantaceae)] clade as a sister group to Costaceae. The cp genome data generated in this study provide valuable insights for developing molecular markers, resolving taxonomic classifications, and advancing phylogenetic and population genetic studies in *Canna* species.

## 1. Introduction

*Canna*, or canna lily, native to the American tropics, is a well-known ornamental plant valued for its brilliantly colored flowers and large tropical foliage. It is widely used in landscapes, gardens, parks, and patios [1,2]. *Canna* is the only genus in the family Cannaceae, which belongs to the order Zingiberales. The genus *Canna* comprises about 51 species, 10 of which are native to America. *C. glauca*, *C. indica*, *C. iridiflora*, *C. warscewiczii*, and *C. flaccid* are recognized as the basal species responsible for the origin of garden cannas [1].

*Canna* is also used in constructed wetlands for water and wastewater treatment due to its high biomass production, rapid growth, and fibrous root structure. The aerenchyma in *Canna* delivers oxygen to the rhizosphere, facilitating bacterial nitrification processes [3,4,5]. In addition to removing environmental nutrients, Canna-planted constructed wetlands efficiently eliminate toxic contaminants such as fluoride, heavy metals, pesticides, pharmaceuticals, and industrial chemicals [3,4,5,6,7,8]. *Canna* root starch has long been used in the food industry, e.g., bakery [9,10]. It has been reported that 49.07% of *Canna* root starch is classified as high-amylose starch [10]. Additionally, *Canna* contains various phytochemicals and exhibits antibacterial, antiviral, anti-inflammatory, analgesic, antioxidant, and other medicinal properties [11,12].

As an ornamental plant valued for its large foliage, *Canna* exhibits leaves in various colors, including green, ruby, purplish, and multicolored forms [2]. Chlorophyll in chloroplasts (cp) is the primary factor influencing leaf color; therefore, chloroplast development and differentiation are crucial for foliage ornamental plants [13,14]. In *Hydrangea macrophylla* var. *maculata*, irregular chloroplast development results in silver-white leaf coloration [15]. In *Liquidambar formosana*, a reduction in chloroplast number and size contributes to leaf color changes under cold stress [16]. Cp, which possess their own genome independent of the nuclear genome, are semi-autonomous organelles primarily responsible for photosynthesis in plants [17,18]. Additionally, chloroplast function influences plant growth and stress tolerance, both of which are critical for phytoremediation [4,19,20]. In higher plants, Cp also participate in various biological processes, including metabolite synthesis and the assimilation of sulfur and nitrogen. These functions not only affect plant growth but also play a key role in plant stress tolerance [17,21].

**Figure 1:**
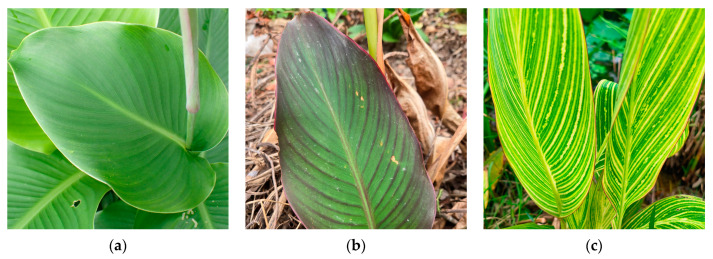
Leaf color phenotypes of three *Canna* species in this study (**a**) *C. edulis* L.; (**b**) *C. warscewiezii* A. Dietr.; (**c**) *C. generalis* ‘Striata’.

Due to its independent genome, not all cp proteins are encoded in the nuclear genome [18]. The cp genome encodes many key proteins involved in photosynthesis and other metabolic processes, highlighting its importance in studying plant light systems, leaf color, and environmental interactions [17,22]. Additionally, cp genomes are commonly used in evolutionary and comparative genomic studies due to their maternal inheritance and highly conserved structure [23,24]. Understanding plant origins and evolution can also aid plant breeding [25]. The difficulty in germinating *Canna* seeds has limited hybridization efforts. Cp genomes may serve as promising targets for genome editing in plant breeding [1,26]. Advancements in high-throughput sequencing and algorithm innovations have made cp genome assembly and whole-cp-genome-based phylogenomics more accessible and cost-effective [23]. However, despite *Canna* being a well-known ornamental plant genus with applications in landscaping, medicine, and phytoremediation, only three complete cp genomes have been sequenced and assembled: *C. indica* (MK561603), *C. edulis* (MK561602, MN832865) [27]. In this study, we selected three *Canna* species with different leaf colors—*C. edulis* L. (green leaves), *C. warscewiezii* A. Dietr. (purplish leaves), and *C. generalis* ‘Striata’ (green leaves with yellow stripes)—to investigate potential relationships between cp genome structures and leaf coloration (Figure 1). Four different cp genomes from these species were sequenced using high-throughput sequencing, and the complete cp genome sequences were assembled. Their genomic structures were characterized, and phylogenetic analyses were conducted. Given the multicolored leaves of *C. generalis* ‘Striata’ cp DNA was separately isolated, sequenced, and analyzed from both the green and golden leaf regions. Additionally, the assembled cp genomes were compared with related species in the Zingiberales order to further explore the phylogenetic position of Cannaceae within Zingiberales. This study primarily characterizes the cp genome structure of *Canna* species and explores their phylogenetic position within Zingiberales. The findings provide valuable insights for further research on Canna phylogenetic classification, molecular marker development, chloroplast gene discovery, and functional genomics.

## 2. Materials and Methods

### 2.1. Plant Material, DNA Extraction and Sequencing

Canna species with different leaf colors (green, purple, and multicolored) were selected for this study. Fresh leaves were collected from *C. edulis*, *C. warscewiezii*, and *C. generalis* ‘Striata’, which were deposited at the Institute of Botany, Jiangsu Province, and the Chinese Academy of Sciences. For *C. generalis* ‘Striata’, both the green and golden parts were sampled. Total leaf DNA was extracted using the EZgene^TM^ SuperFast Plant Leaves DNA Kit (Biomiga, San Diego, CA, USA). DNA quality was assessed via spectrophotometry and agarose gel electrophoresis. An average of 350 bp paired-end libraries were prepared using the Illumina TruSeq DNA Sample Prep Kit (Illumina Inc., San Diego, CA, USA) and sequenced on an Illumina NovaSeq 6000 platform following the manufacturer’s protocol.

### 2.2. Genome Assembly and Annotation

Raw reads were filtered using fastp [28], retaining those with <5% unidentified nucleotides and >50% of bases with a quality score > 20 as high-quality reads. The high-quality reads were then aligned to a reference cp genome database constructed by Genepioneer Biotechnologies (Nanjing, China) to extract cp-like reads using Bowtie2 [29]. The extracted reads were assembled into contigs and scaffolds using the de novo assembler SPAdes v3.15.5 [30] and SSPACE 2.1.1 [31], followed by gap filling with Gapfiller v1.11 [32]. Coding sequences (CDS), rRNA, and tRNA were predicted using Prodigal v2.6.3 [33], HMMER 3v.3.2 [34], and ARAGORN v1.2.41 [35], respectively. The genome map was visualized using OrganellarGenomeDRAW (OGDRAW) [36] [https://chlorobox.mpimp-golm.mpg.de/OGDraw.html, accessed on 17 August 2024] and the R package Chloroplot v0.2.4 [37].

### 2.3. The Relative Synonymous Codon Usage Analysis (RSCU), Simple Sequence Repeats (SSR) Prediction

Unique CDS were filtered using Perl scripts developed by Genepioneer Biotechnologies (Nanjing, China). The RSCU value was calculated as the ratio of the observed frequency to the expected frequency of a particular codon. Synonymous codon preference was categorized into four models: high preference (RSCU > 1.3), moderate preference (1.2 ≤ RSCU ≤ 1.3), low preference (1.0 < RSCU < 1.2), and no preference (RSCU ≤ 1.0) [38]. Simple sequence repeats (SSRs) were identified using MISA v2.1 [39] with the following search parameters: mono-nucleotide units appearing at least 8 times, di- nucleotide units 5 times, tri-nucleotide units 3 times, and tetra-, penta-, and hexa-nucleotide units 3 times each.

### 2.4. Sequence Divergence Analyses of the Four Canna Cp Genomes

Using the cp genome of *C. edulis* (MN832865) as a reference, the four *Canna* complete cp genomes were compared using mVISTA in Shuffle-LAGAN mode (https://genome.lbl.gov/vista/mvista/submit.shtml, accessed on 17 August 2024) [40,41]. IRscope v0.1.R was used to analyze variations in the LSC/IRb/SSC/IRa region borders [42].

### 2.5. Phylogenetic Analysis

A phylogenetic tree was constructed based on the four cp genome datasets and 30 additional Zingiberales cp genomes downloaded from GenBank, with *Zea mays* L. (NC001666) as the outgroup. Genome sequence alignment was performed using MAFFT v7.453 [43,44]. Additionally, phylogenetic trees were generated using MAFFT and MEGA11, applying the Maximum Likelihood method and Tamura-Nei model with 1000 bootstrap replicates [45,46].

## 3. Results

### 3.1. Genome Assembly and Structure of the Four Canna Species Cp Genomes

Four different cp genomes of *Canna* species (*C.* with green leaves, *C. warscewiezii* A. Dietr. with purplish leaves, and *C. generalis* ‘Striata’ with green leaves and golden stripes) were sequenced. Then, the complete circular chloroplast genomes of them were assembled. The assembled cp genomes were similar in size: *C. edulis* (164,479 bp), *C. warscewiezii* (164,509 bp), *C. generalis* ‘Striata’ (green, 164,427 bp), and *C. generalis* ‘Striata’ (yellow, 164,479 bp). Each cp genome exhibits a typical quadripartite structure, consisting of a large single-copy (LSC) region, a small single-copy (SSC) region, and a pair of inverted repeat (IR) regions (Table 1). The overall GC content ranges from 36.23% to 36.25%, with the IR regions having the highest GC content (42.36–42.48%) and the SSC region the lowest (30.14–30.23%).

### 3.2. Gene Annotation of the Four Canna Species Cp Genomes

**Figure 2:**
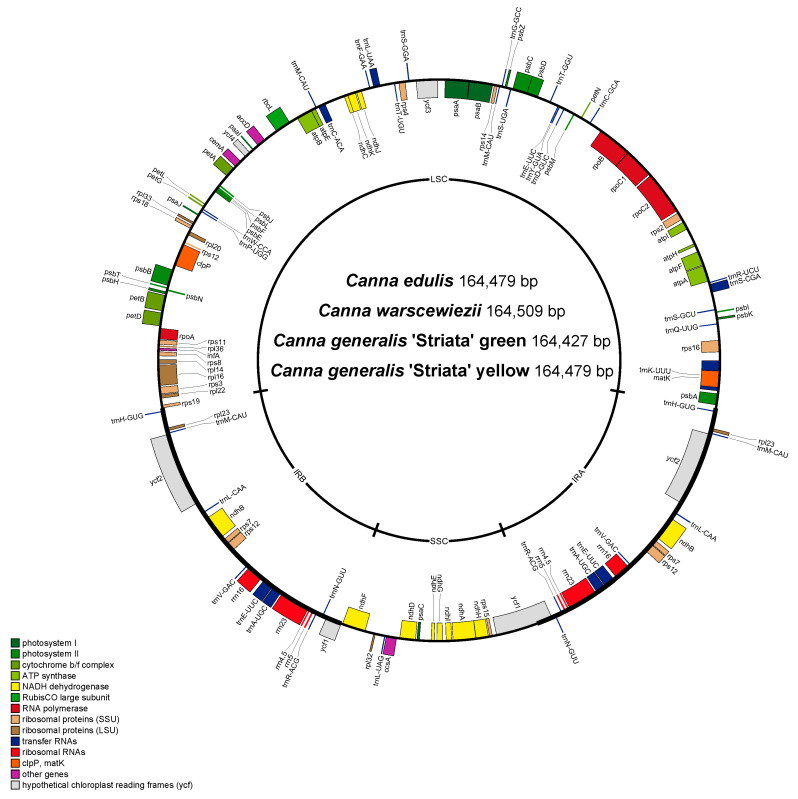
Gene map of the four complete chloroplast genomes. Genes shown above are transcribed clockwise, and genes below the circle are transcribed counterclockwise. Genes belonging to the same functional groups are color coded.

The predicted genes of the four cp genomes were assembled and annotated (Figure S1). Notably, all four genomes share an identical structure. Each genome contains 130 predicted genes, including 110 unique genes (78 protein-coding genes, including four of unknown function, four rRNAs, and 28 tRNAs), 18 duplicated genes (six protein-coding genes, including two of unknown function, four rRNAs, and eight tRNAs) located in the IR regions, and two *trnM-CAU* genes in the LSC region (Figure 2, Table 1). Most genes lack introns, except for nine protein-coding genes and six tRNA genes, which contain a single intron, and three protein-coding genes, which contain two introns. A total of 18 genes with introns are shared with many other plants [47]. Gene function analysis classified the genes into four categories: 44 associated with photosynthesis, 56 involved in self-replication, six with other functions, and four of unknown function (Table 2). *trnK-UUU* has the longest intron (2765 bp), which contains *matK*. This finding is consistent with those in the cp genomes of *Ilex*, *Zingiber*, *Cymbidium*, and *Forsythia* [48].

### 3.3. Codon Preference Analysis

**Figure 3:**
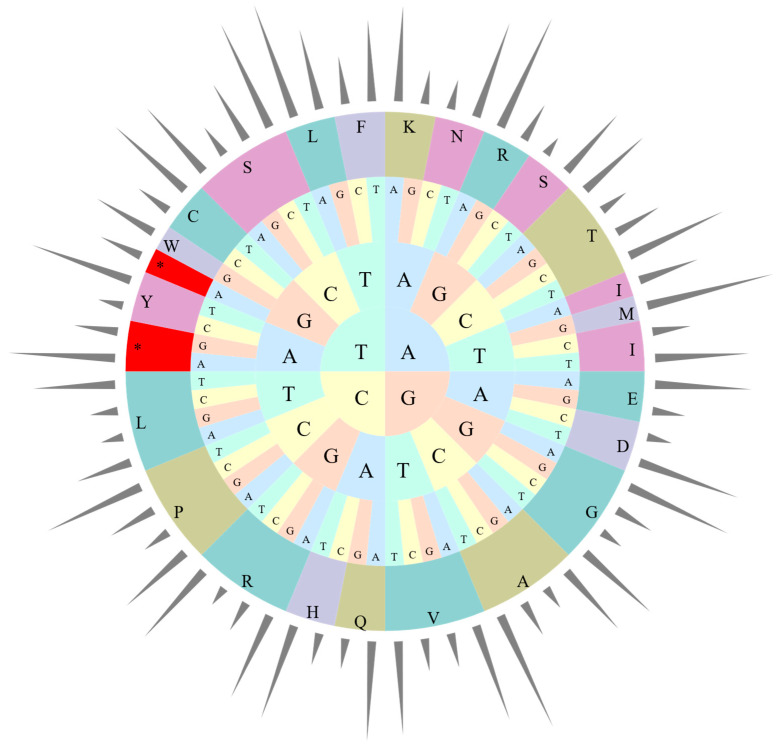
Codon content of 20 amino acids in all cp protein-coding genes of *Canna* species. The bars out of the circle indicate RSCU, while the pie inside indicates the codons. From inside to outside, the first to third circles represent three bases of each codons with different bases in different colors. The fourth circle represents different amino acids, while different colors were used to distinguish different amino acids. *: Termination codon.

The four cp genomes in this study exhibit the same codon preference pattern, with three termination codons and 63 codons encoding amino acid sequences in genes. Only tryptophan encoding shows no codon preference (Figure 3, Table S1). Among all codons, 31 preferred codons were identified—30 encoding 18 amino acids and one being a stop codon. An RSCU value of 1.0 < RSCU < 1.2 indicates a weak preference, 1.2 ≤ RSCU ≤ 1.3 represents a moderate preference, and RSCU > 1.3 signifies a strong preference [38]. A total of 66 codon types (25,987 codons in total) encoded 20 different amino acids, with 31 codons having RSCU > 1. Three codons showed weak preference (9.68%), five exhibited moderate preference (16.13%), and 23 displayed strong preference (74.19%). Similarly to many other cp genomes, most preferred codons ended with A or T, except for TTG [48].

### 3.4. Repeat Structure and SSR Analysis

**Figure 4:**
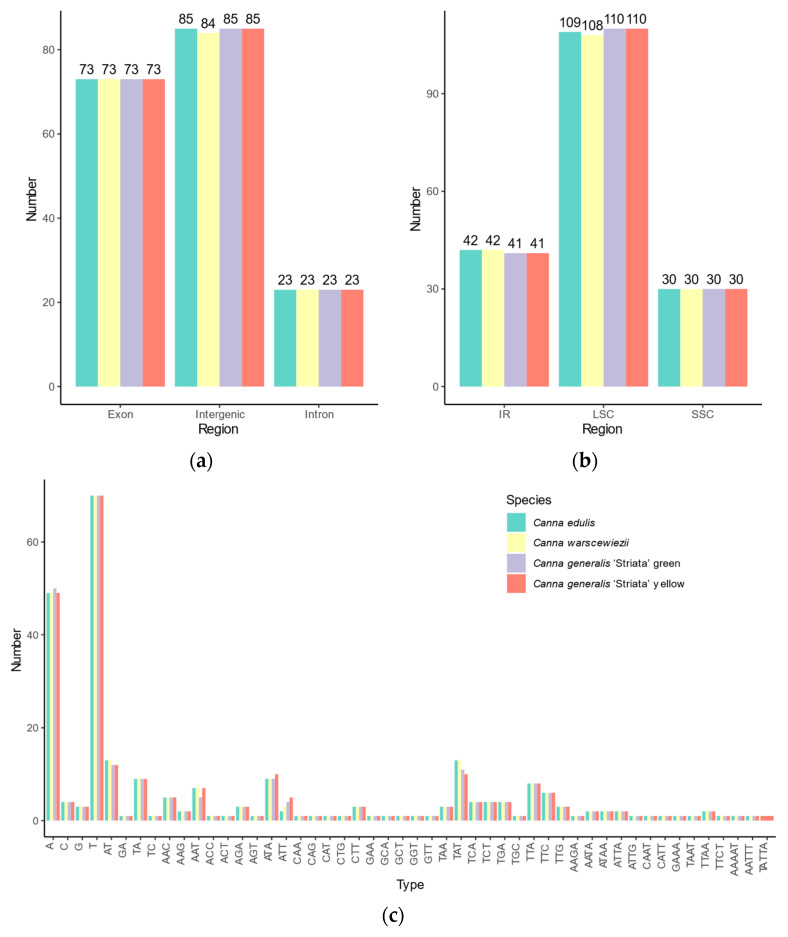
Distribution of SSR Types in *Canna* species cp genomes. SSR numbers in (**a**) intergenic regions, introns, and coding regions; (**b**) different genomic regions; (**c**) different repeat class types.

Simple sequence repeats (SSRs) loci in the four cp genomes were identified, revealing similar SSR distribution patterns (Figure 4). A total of 181 SSR loci were identified in *C. edulis* and *C. generalis* ‘Striata’, whereas *C. warscewiezii* had 180, one fewer than the other three cp genomes. Among the SSRs, 43, 44, 44, and 43 complex SSRs with multiple repeats were found in *C. edulis*, *C. warscewiezii*, *C. generalis* ‘Striata’ (green part), and *C. generalis* ‘Striata’ (yellow part, respectively. The number of complex SSRs was significantly higher than in other plant species such as *Ilex* and *Eremochloa* species [48,49]. Among perfect SSRs, 84, 83, 84, and 84 mononucleotide repeats were identified in *C. edulis*, *C. warscewiezii*, *C. generalis* ‘Striata’ (green part), and *C. generalis* ‘Striata’ (yellow part, respectively. The number of dinucleotide SSRs was 7, 6, 6, and 7. The trinucleotide and tetranucleotide SSRs were consistent across cp genomes, with 42 trinucleotide and five tetranucleotide repeats each. Most mononucleotide repeats consisted of polyadenine (poly A) and polythymine (poly T), with only a few polyguanine (poly G) and polycytosine (poly C) repeats (seven in each cp genome). All dinucleotides in the four cp genomes were AT/TA repeat motifs, while GA/TC repeat motifs occurred only in complex SSR loci. Pentanucleotide repeats were identified exclusively in complex SSR loci, with one AAAAT and one AATTT repeat motif in all four cp genomes and one TATTA repeat motif in *C. generalis* ‘Striata’ (yellow part). SSR distribution varied across genomic regions. Each cp genome contained 73 SSRs in introns and 23 in coding regions. In intergenic regions, 85 SSRs were detected in *C. edulis* and *C. generalis* ‘Striata, whereas *C. warscewiezii* had 84. More than half of the SSRs were located in LSC regions (109, 108, 110, and 110 in each cp genomes, respectively).

**Figure 5:**
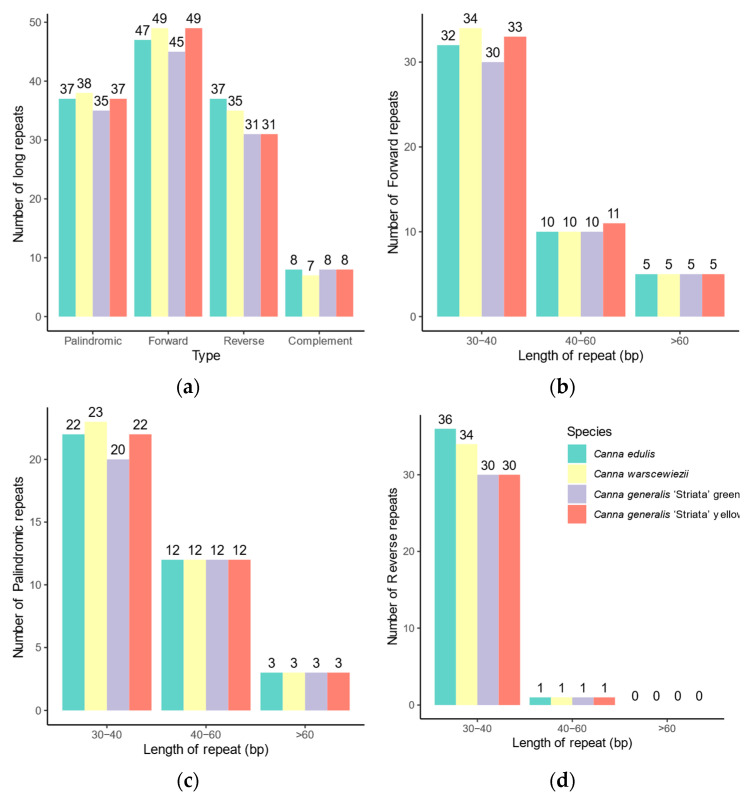
Distribution of long repeat sequences in the four *Canna* species cp genomes. (**a**) Number of the long repeats in different types; (**b**) Length distribution of forward repeats; (**c**) Length distribution of palindromic repeats; (**d**) Length distribution of reverse repeats.

The long-dispersed repeats exhibit greater differences among the four cp genomes (Figure 5) than SSRs. Long repeats can be classified into four types based on direction and complementarity: forward, reverse, complement, and palindromic. In the cp genomes of *C. edulis* and *C. warscewiezii*, 129 long repeats were identified. The *C. generalis* ‘Striata’ yellow part cp genome contains 125 long repeats, while the green part cp genome has only 119. More than 40 repeats in each cp genome are forward repeats (ranging from 45 to 49), most of which are located in the LSC regions (Table S2). The *C. generalis* ‘Striata’ cp genomes contain fewer reverse repeats (31 in each) compared to *C. edulis* and *C. warscewiezii* (37 and 35, respectively). Similarly to cp genomes in some other plants, most long repeats are 30–40 bp in length [22,48,50].

### 3.5. Comparative Chloroplast Genome Analysis

**Figure 6:**
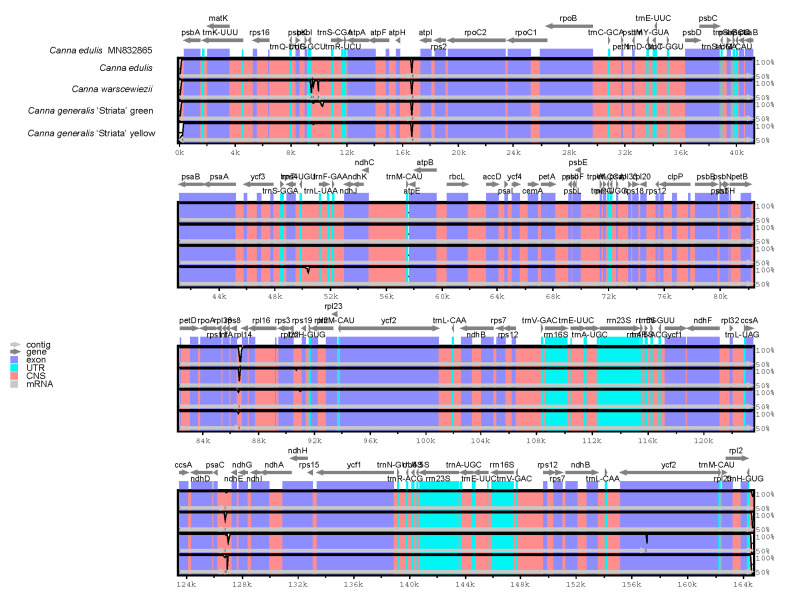
Comparisons between four *Canna* species cp genomes and published cp genome of *C. edulis* (MN832865). Gray arrows above represent gene orientation. Dark blue bars represent exons. Light blue bars represent UTRs. Pinks bars represent conserved non-coding sequences. The vertical axis indicates the percentage of identity. White peaks represent sequence variation among the cp genomes.

The entire cp genomes of *C. edulis*, *C. warscewiezii*, and *C. generalis* ‘Striata’ were compared with the published *C. edulis* cp genome (MN832865) as a reference using mVISTA (Figure 6). White peaks in the figure indicate sequence variation among *Canna* species. The results reveal a high degree of sequence conservation between the four cp genomes and the published *C. edulis* cp genome. Sequence variations were mainly found in non-coding regions rather than coding regions, with only one variable site detected in the exons of *ycf2*. Although the four materials exhibit different leaf colors, only minor variations were observed between their cp genomes, suggesting that cp genomes have a limited effect on leaf color. However, these variations could be useful for developing DNA markers for *Canna* species identification or phylogenetic research.

### 3.6. Expansion and Contraction of IRs

**Figure 7:**
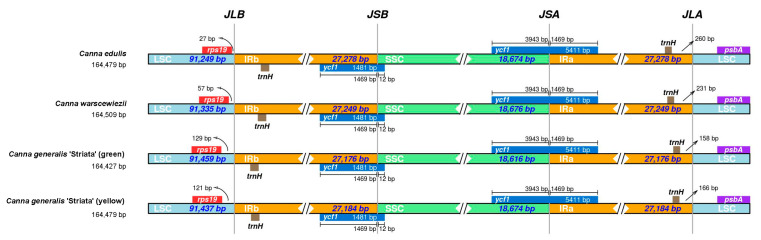
Comparison of the IR region boundaries among the four *Canna* species cp genomes. LSC, IR, and SSC regions were indicated by light blue, yellow, and green. Boxes above and below the bars represent denoted genes. The base length between genes and boundaries are labeled (bp). Different colors represent different regions.

The comparison of the IR boundaries and their adjacent genes in the four cp genomes is presented in Figure 7. Overall, the junctions are highly conserved across all four cp genomes, particularly for JSB (SSC/IRb) and JSA (SSC/IRa). The pseudogenes located at JSA and JSB are both *ycf1*, and the distances between the gene boundaries and the junctions are identical among all four cp genomes. The distances between *ycf1* boundaries and JSB are 1469 bp and 12 bp, while for JSA, they are 3942 bp and 1469 bp. The pseudogene closest to JLB is *rps19*, and the distances between them vary among the four cp genomes. The shortest distance is 27 bp in the *C. edulis* cp genome, 57 bp in the *C. warscewiezii* cp genome, and over 120 bp in both *C. generalis* ‘Striata’ cp genomes. The *trnH* pseudogenes are located within the IR regions in all four cp genomes. The distance between *trnH* and the LSC boundary in the *C. edulis* cp genome is 260 bp, which is 29 bp longer than in the *C. warscewiezii* cp genome. In *C. generalis* ‘Striata’, the distances are shorter: 150 bp for the green part and 166 bp for the yellow part.

### 3.7. Phylogenetic Relationship Analysis of Cannaceae

**Figure 8:**
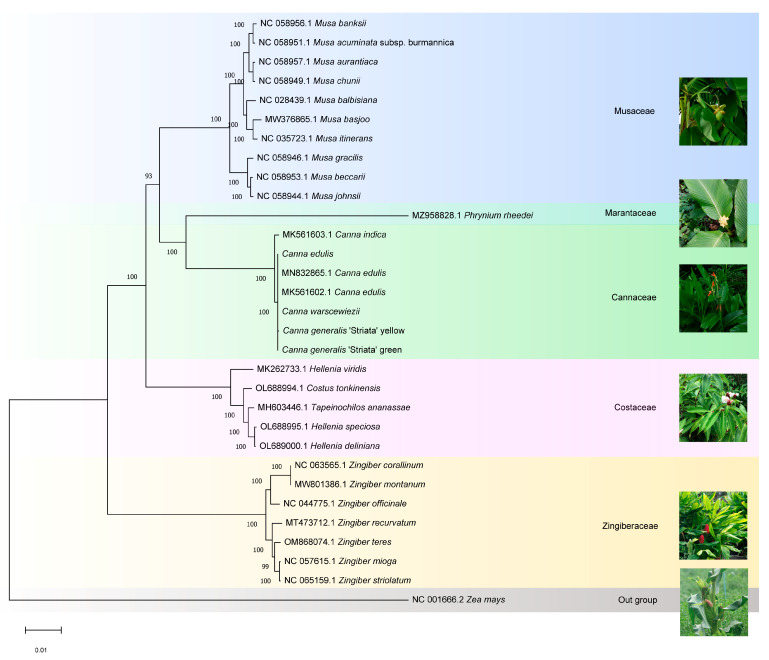
Phylogenetic tree of the Zingiberales species based on the complete cp genome data constructed using the Maximum likelihood (ML) method and Tamura-Nei model. 31 species were used to reconstruct a phylogenetic tree. *Zea mays* was used as the outgroup.

The phylogenetic relationships of *Canna* species and Cannaceae were determined. The four cp genomes generated in this study, along with the published cp genome sequences of 31 Zingiberales species, were aligned using MAFFT. *Zea mays* (Gramineae) was set as the outgroup. A phylogenetic tree was constructed using the Maximum Likelihood method and the Tamura-Nei model with 1000 bootstrap replicates in MEGA11 (Figure 8). Species within each family clustered into distinct clades. Cannaceae contains only one genus, *Canna*, in which *C. edulis* (MK561602, MN832865, and the genome generated in this study), *C. warscewiezii*, and *C. generalis* ‘Striata’ formed a closely related group. In contrast, *C. indica* (MK561603) was placed outside this group, suggesting that cultivars with different leaf colors may have been bred from *C. edulis*. According to the APG IV system, Cannaceae is classified under the order Zingiberales [51] and is closely related to Marantaceae, consistent with previous research findings [52]. However, the relationships between Cannaceae and other ginger families (Costaceae and Zingiberaceae) were not well supported. It is generally believed that Costaceae and Zingiberaceae form a group close to Cannaceae and Marantaceae. However, in the phylogenetic tree generated in this study based on cp genomes, Cannaceae and Marantaceae were more closely related to Musaceae than to Costaceae or Zingiberaceae. This result aligns with Zhu et al.’s findings using the same cp genome-based approach [27,53]. Additionally, Costaceae was found to be more closely related to Cannaceae than Zingiberaceae.

## 4. Discussion

### 4.1. The Structures and Gene Identification in Canna Species Cp Genomes

In land plants, cp genomes are highly conserved in both size and structure. The cp genomes of most land plants range from 120 to 160 kb in length; however, genome size varies from 15,553 bp in *Asarum minus* to 521,168 bp in *Floydiella terrestris* [47,54]. The cp genome structures of vascular plants are more dynamic than those of nonvascular plants [47]. In this study, the cp genomes of the four *Canna* species are approximately 164.5 kb in length, similar to other reported *Canna* species cp genomes [27] and Zingiberales cp genomes, which range from 161 kb in *Zingiber montanum* to 173 kb in *Musa basjoo*. All four *Canna* cp genomes exhibit a circular structure with a typical quadripartite organization, featuring LSC and SSC regions separated by a pair of IRs, similar to most angiosperms. The expansion and contraction of IRs are key mechanisms driving cp genome size variation. IRs in seed plants are typically larger (20–30 kb) than those in other land plants (10–15 kb) [47]. The IR sizes in the four *Canna* species range from 27.18 kb (*C. generalis* ‘Striata’) to 27.28 kb (*C. edulis*), which are larger than those in many Gramineae species (20–23 kb). However, the LSC and SSC regions in *Canna* species are also larger than those in Gramineae species [49,55,56]. Additionally, the four cp genomes share similar GC content across the complete genome and different regions.

In land plants, each cp genome typically contains about 80 protein-coding genes, 4 rRNAs, and 30 tRNAs [47,57,58]. The gene structure of the four cp genomes is identical in both gene number and function, with 130 predicted genes categorized into the same functional groups. In these cp genomes, IR expansion has relocated *ycf2* and *trnH* from the LSC into the IR, similar to the IR expansion observed in *Acorus* species cp genomes [47,59]. Interestingly, in the four Canna species cp genomes, *trnM-CAU* is not only duplicated in the IR regions but also present in the LSC region with two copies. This duplication pattern, also found in a previously reported *Canna* cp genome [27], is uncommon in other plant cp genomes such as those of *Ilex* [48], Gramineae [49], *Trifolium* [22], or *Lespedeza* [60]. As ATG is the start codon for methionine (M), the multiple copies of *trnM-CAU* in *Canna* species may reflect dynamic translation activity in their chloroplasts.

### 4.2. Repeat Sequences in Canna Species Cp Genomes

Different types of repeat sequences are major components of plant genomes, accounting for up to 90% of genome size. These repeat sequences are either distributed throughout the genome or confined to specific regions [61,62]. They play multiple roles, including gene expression regulation, genome stability, recombination, and chromatin modulation [63]. In this study, more than 100 dispersed repeats were identified in each of the cp genomes, with most located in the LSC regions, similar to other plant cp genomes such as *Ilex dabieshanensis* [48] and *Eremochloa ophiuroides* [64]. A high content of dispersed repeats in the LSC may be a common feature of plant cp genomes.

SSRs are short sequences arranged in tandem repeats of 1 to 6 nucleotide motifs. They are highly abundant and randomly dispersed throughout nuclear and plastid genomes [65]. Due to their high polymorphism and abundance, SSRs are commonly used for marker-assisted selection (MAS) and map-based cloning [66,67]. Previous research has shown that SSRs provide sufficient polymorphism among cp genomes [68]. However, in the four *Canna* cp genomes, SSRs exhibited similar patterns, with nearly identical numbers (180–181) and repeat type. In contrast, long repeats showed greater variation in type, number, and distribution in types, numbers, and distributions. *Canna* breeders could utilize long repeats as molecular markers through techniques such as PCR or Sanger sequencing due to their convenience and low cost [66,67]. Most SSR motifs in the four cp genomes are rich in adenine (A) and thymine (T), consistent with previously reported cp SSRs [22,48,64].

### 4.3. Phylogenetic Analysis via Cp Genomes

Over the past three decades, plant cp genomes have been widely used in evolutionary and phylogenetic research due to their sequence conservation, small and simple genome, and limited horizontal gene transfer [69]. To date, thousands of complete cp genome sequences from over 2000 species have been uploaded to GenBank [47], providing valuable resources for studying phylogenetic relationships in plants [69]. Newly sequenced cp genomes will continue to enhance our understanding of relationships among various orders, families, and genera. In this study, the four cp genomes provide new sequences for research on the evolution of the Cannaceae family and the Zingiberales order. The phylogenetic analysis suggests a clade of (Musaceae (Cannaceae + Marantaceae)) as a sister group to Costaceae, differing from the clade ((Cannaceae + Marantaceae) + Costaceae + Zingiberaceae)) identified using multiplexed exon capture technology, which strongly supports the monophyly of the ginger families [52]. However, similar results have also been reported in previous studies based solely on cp genomes [27]. Conflicts between phylogenetic relationships inferred from cp genomes and those based on nuclear genes or genomes are common. These conflicts may provide insights into significant evolutionary events such as ancient hybridization, polyploidy, and incomplete lineage sorting [69]. Only a few complete cp genomes of the Cannaceae family have been sequenced and assembled, far fewer than those of Musaceae or Zingiberaceae (available in GenBank). Additionally, compared to cp genomes, mitochondrial genomes (mt genomes) vary greatly in both size and structure. Integrating mt genome information could offer new perspectives on taxonomy [70]. However, mt genome data for Zingiberaceae remain limited. To clarify relationships within the Zingiberales order, more cp, mt, and nuclear genomes from the Cannaceae family should be sequenced and uploaded, given the ease of obtaining complete sequences using next-generation sequencing technology [23,47].

## 5. Conclusions

The complete cp genomes of four *Canna* species with different leaf colors were sequenced, assembled, and analyzed in this study. All four cp genomes exhibited the same gene prediction pattern and codon preference. Each cp genome contained 130 predicted genes, including 110 unique genes, 18 duplicated genes in the IR regions, and 2 *trnM-CAU* genes in the LSC region. Codon usage showed a bias toward A/T endings, similar to other plants. Comparative cp genome analyses showed high conservation among the four cp genomes. Dispersed long repeats exhibited greater polymorphism than SSRs, suggesting their potential utility in *Canna* breeding. These findings further highlight the conserved nature of plant cp genomes. Phylogenetic analysis confirmed the close relationship between Cannaceae and Marantaceae. However, Musaceae was found to be more closely related to Cannaceae and Marantaceae than to Costaceae or Zingiberaceae, providing new insights into their evolutionary history. The cp genome data generated in this study are valuable for germplasm identification, taxonomic clarification, phylogenetic resolution, and genetic improvement of Cannaceae. Additionally, integrating these data with other omics approaches, such as transcriptomics and metabolomics, could provide a more comprehensive understanding of *Canna* biology and its potential for genetic enhancement.

## Figures and Tables

**Table 1:** Summary of the four Canna species cp genomes.

Genome Features	*C. edulis*	*C. warscewiezii*	*C. generalis* ‘Striata’ Green	*C. generalis* ‘Striata’ Yellow
Genome size(bp)/ GC content (%)	164,479/36.24	164,509/36.23	164,427/36.25	164,479/36.24
LSC size (bp)/GC content (%)	91,249/33.82	91,335/33.80	91,459/33.77	91,437/33.77
SSC size (bp)/GC content (%)	18,674/30.15	18,676/30.15	18,616/30.23	18,674/30.14
IRa size (bp)/GC content (%)	27,278/42.36	27,249/42.39	27,176/42.49	27,184/42.48
IRb size (bp)/GC content (%)	27,278/42.36	27,249/42.39	27,176/42.49	27,184/42.48
Total gene number	130	130	130	130
mRNAs	84	84	84	84
tRNAs	38	38	38	38
rRNAs	8	8	8	8
pseudogenes	0	0	0	0
Genes duplicated in IRs	18	18	18	18

**Table 2:** Summary of gene annotation for the four cp genomes.

Category	Gene Group	Gene Name
Photosynthesis	Subunits of photosystem I	*psaA*, *psaB*, *psaC*, *psaI*, *psaJ*
	Subunits of photosystem II	*psbA*, *psbB*, *psbC*, p*sbD*, *psbE*, *psbF*, *psbH*, *psbI*, *psbJ*, *psbK*, *psbL*, *psbM*, *psbN*, *psbT*, *psbZ*
	Subunits of NADH dehydrogenase	ndhA*, ndhB*(2), *ndhC*, *ndhD*, *ndhE*, *ndhF*, *ndhG*, *ndhH*, *ndhI*, *ndhJ*, *ndhK*
	Subunits of cytochrome b/f complex	*petA*, petB*, petD*, *petG*, *petL*, *petN*
	Subunits of ATP synthase	*atpA*, *atpB*, *atpE*, atpF*, *atpH*, *atpI*
	Large subunit of rubisco	*rbcL*
	Subunits photochlorophyllide reductase	-
Self-replication	Proteins of large ribosomal subunit	*rpl14*, rpl16*, *rpl20*, *rpl22*, *rpl23*(2), *rpl32*, *rpl33*, *rpl36*
	Proteins of small ribosomal subunit	*rps11*, rps12**(2), *rps14*, *rps15*, rps16*, *rps18*, *rps19*, *rps2*, *rps3*, *rps4*, *rps7*(2), *rps8*
	Subunits of RNA polymerase	*rpoA*, *rpoB*, rpoC1*, rpoC2*
	Ribosomal RNAs	*rrn16*(2), *rrn23*(2), *rrn4.5*(2), *rrn5*(2)
	Transfer RNAs	trnA-UGC*(2), trnC-ACA*, *trnC-GCA*, *trnD-GUC*, *trnE-UUC*, trnE-UUC*(2), *trnF-GAA*, *trnG-GCC*, *trnH-GUG*(2), trnK-UUU*, *trnL-CAA*(2), trnL-UAA*, *trnL-UAG*, *trnM-CAU*(4), *trnN-GUU*(2), *trnP-UGG*, *trnQ-UUG*, *trnR-ACG*(2), *trnR-UCU*, trnS-CGA*, *trnS-GCU*, *trnS-GGA*, *trnS-UGA*, *trnT-GGU*, *trnT-UGU*, *trnV-GAC*(2), *trnW-CCA*, *trnY-GUA*
Other genes	Maturase	*matK*
	Protease	clpP**
	Envelope membrane protein	*cemA*
	Acetyl-CoA carboxylase	*accD*
	c-type cytochrome synthesis gene	*ccsA*
	Translation initiation factor	*infA*
	other	-
Genes of unknown function	Conserved hypothetical chloroplast ORF	*ycf1*(2), *ycf2*(2), ycf3**, *ycf4*

Gene*: Gene with one introns; Gene**: Gene with two introns; Gene(2): Number of copies of multi-copy genes.

## Data Availability

The data that support the findings of this study are openly available in GenBank (www.ncbi.nlm.nih.gov/, accessed on 4 October 2024.), accession number OR502631, OR502632, OR502633, and OR502634.

## Supplementary Materials

The following supporting information can be downloaded at: https://www.mdpi.com/article/10.3390/cimb47040222/s1.

## Abbreviations

The following abbreviations are used in this manuscript:

cp	Chloroplast
MAS	Molecular assistant selection
ML	Maximum likelihood
LSC	Long single-copy
SSC	Short single-copy
SSR	Simple sequence repeat
IR	inverted repeats
