# Supplementary material for: Complete Chloroplast Genome Sequences of Three Canna Species: Genome Characterization, Comparative Analyses, and Phylogenetic Relationships Within Zingiberales
Source: Curr Issues Mol Biol. 2025 Mar 25;47(4):222. doi: 10.3390/cimb47040222 (PMC12026059; doi:10.3390/cimb47040222)
Supplement: Supplementary file 1 [file cimb-47-00222-s001.zip › FigureS1 gene map of 4 cp genomes.pdf]

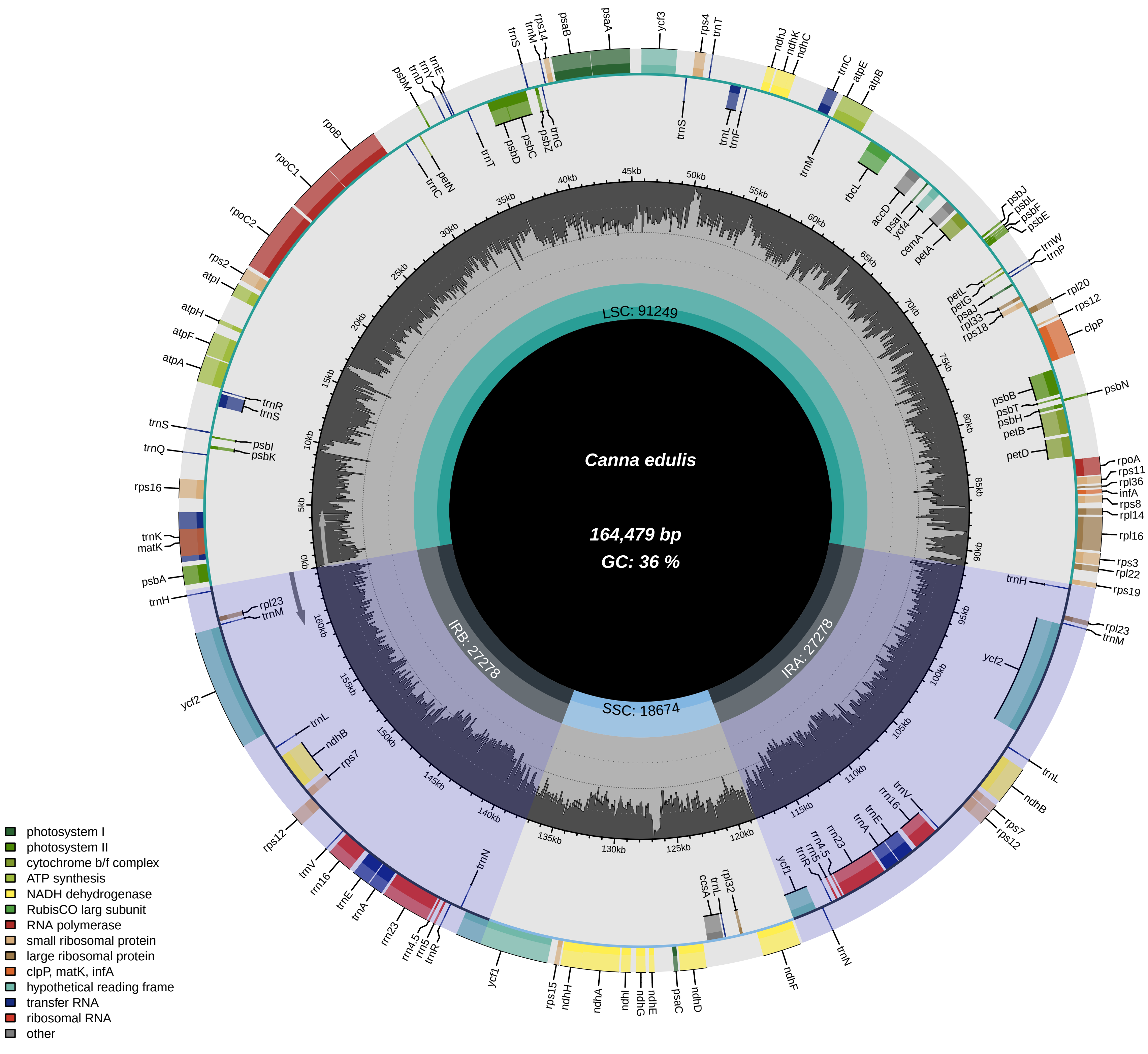

Canna edulis
164,479 bp
GC: 36 %
LSC: 91249
IRA: 27278
SSC: 18674
IRB: 27278
photosystem I
photosystem II
cytochrome b/f complex
ATP synthesis
NADH dehydrogenase
RubisCO larg subunit
RNA polymerase
small ribosomal protein
large ribosomal protein
clpP, matK, infA
hypothetical reading frame
transfer RNA
ribosomal RNA
other

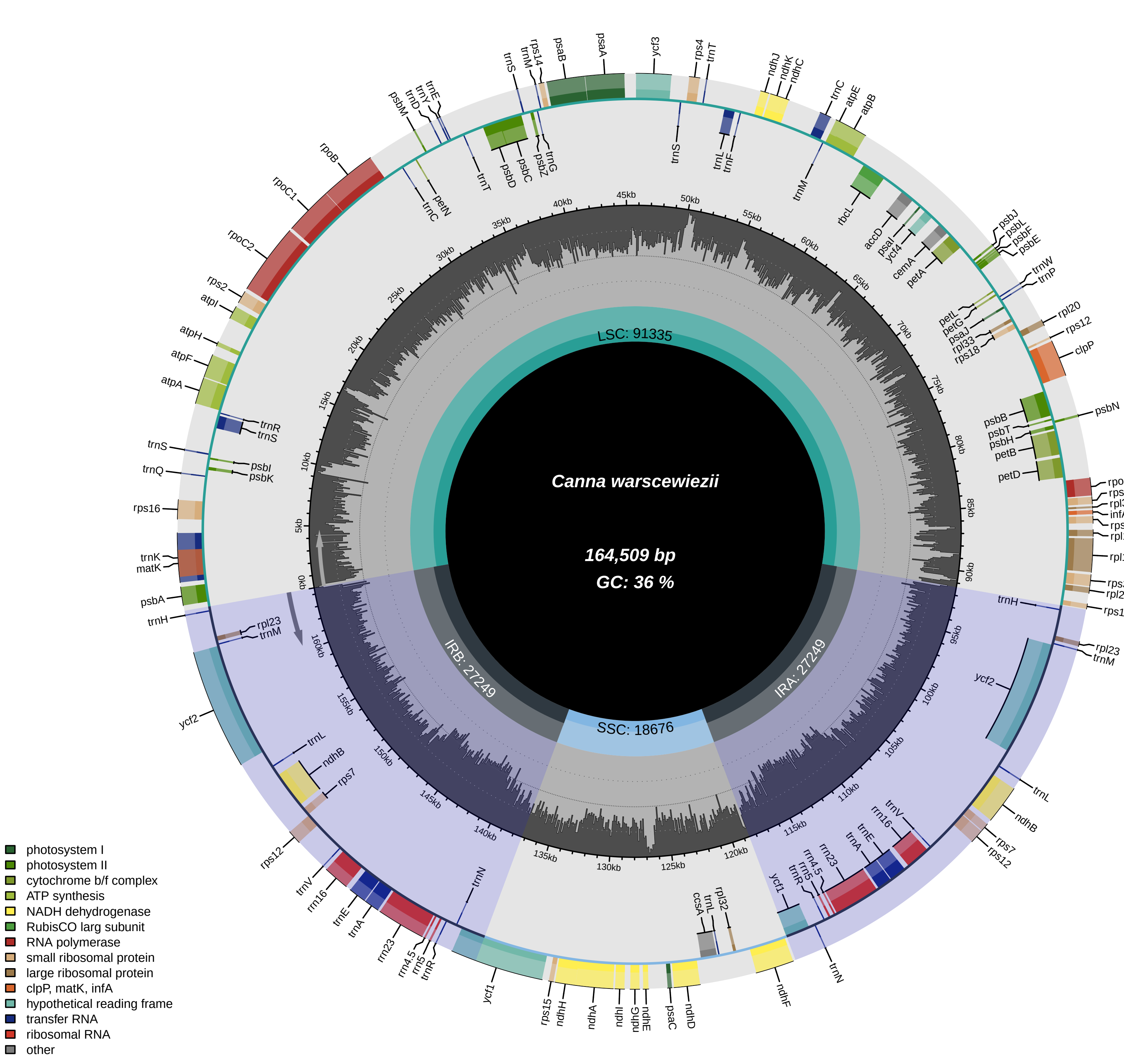
Canna warscewiezii
164,509 bp
GC: 36 %
LSC: 91335
IRA: 27249
SSC: 18676
IRB: 27249
0kb
5kb
10kb
15kb
20kb
25kb
30kb
35kb
40kb
45kb
50kb
55kb
60kb
65kb
70kb
75kb
80kb
85kb
90kb
95kb
100kb
105kb
110kb
115kb
120kb
125kb
130kb
135kb
140kb
145kb
150kb
155kb
160kb
psbA
trnH
trnK
matK
rps16
trnQ
trnS
psbI
psbK
trnR
trnS
atpA
atpF
atpH
atpI
rps2
rpoC2
rpoC1
rpoB
trnC
petN
psbM
trnD
trnY
trnE
trnT
psbD
psbC
psbZ
trnG
trnS
trnM
rps14
psaB
psaA
ycf3
trnS
rps4
trnT
trnL
trnF
ndhJ
ndhK
ndhC
trnM
trnC
atpE
atpB
rbcL
accD
psaI
ycf4
cemA
petA
psbJ
psbL
psbF
psbE
trnW
trnP
petL
petG
psaJ
rpl33
rps18
rpl20
rps12
clpP
psbN
psbB
psbT
psbH
petB
petD
rpoA
rps11
rpl36
infA
rps8
rpl14
rpl16
rps3
rpl22
rps19
trnH
rpl23
trnM
ycf2
trnL
ndhB
rps7
rps12
trnV
rrn16
trnE
trnA
rrn23
rrn4.5
rrn5
trnR
trnN
ycf1
rpl32
trnL
ccsA
ndhF
ndhD
psaC
ndhE
ndhG
ndhI
ndhA
ndhH
rps15
ycf1
trnN
trnR
rrn5
rrn4.5
rrn23
trnA
trnE
rrn16
trnV
rps12
rps7
ndhB
trnL
ycf2
rpl23
trnM
photosystem I
photosystem II
cytochrome b/f complex
ATP synthesis
NADH dehydrogenase
RubisCO larg subunit
RNA polymerase
small ribosomal protein
large ribosomal protein
clpP, matK, infA
hypothetical reading frame
transfer RNA
ribosomal RNA
other

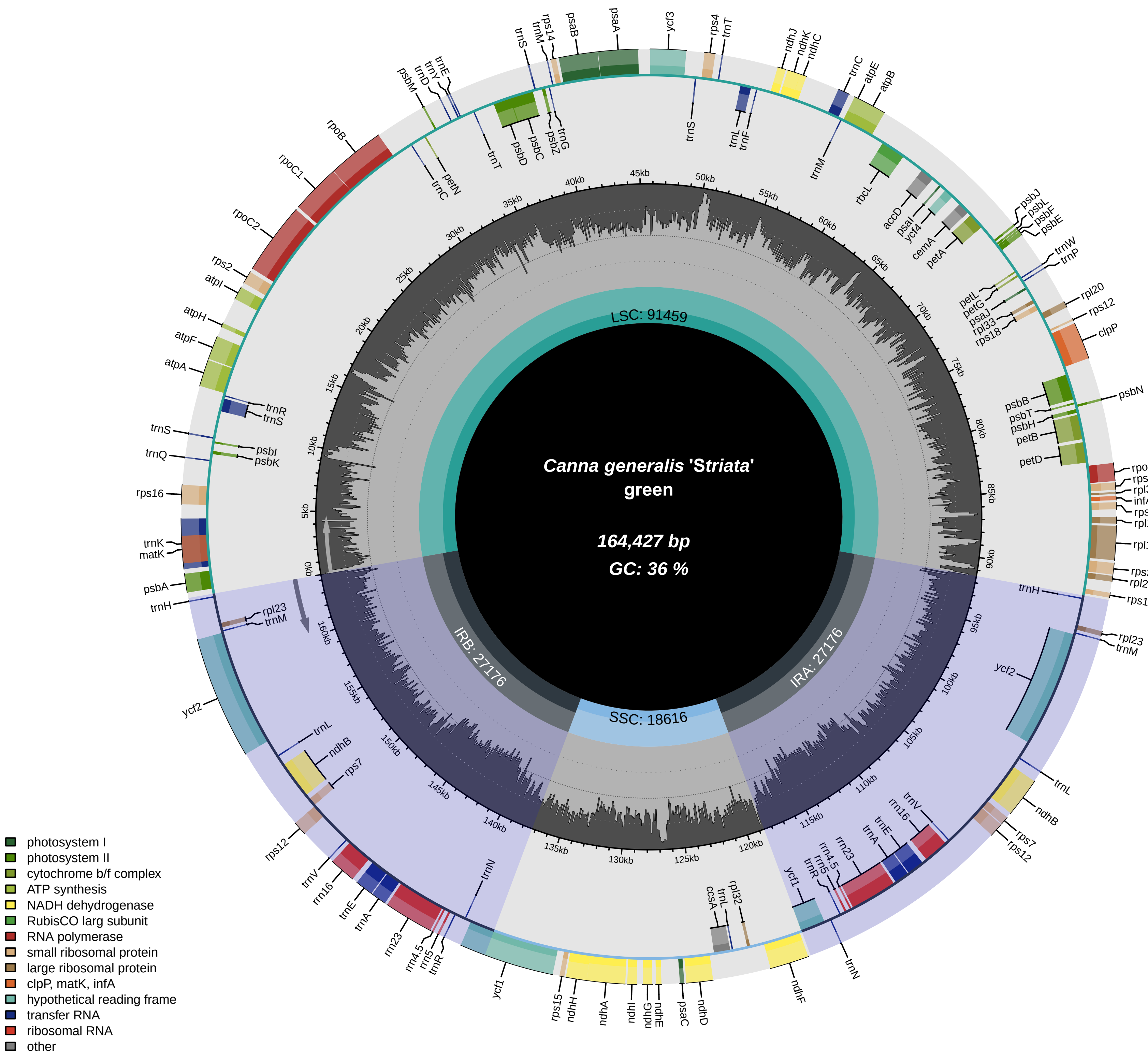
Canna generalis 'Striata'
green
164,427 bp
GC: 36 %
LSC: 91459
IRA: 27176
SSC: 18616
IRB: 27176
photosystem I
photosystem II
cytochrome b/f complex
ATP synthesis
NADH dehydrogenase
RubisCO larg subunit
RNA polymerase
small ribosomal protein
large ribosomal protein
clpP, matK, infA
hypothetical reading frame
transfer RNA
ribosomal RNA
other

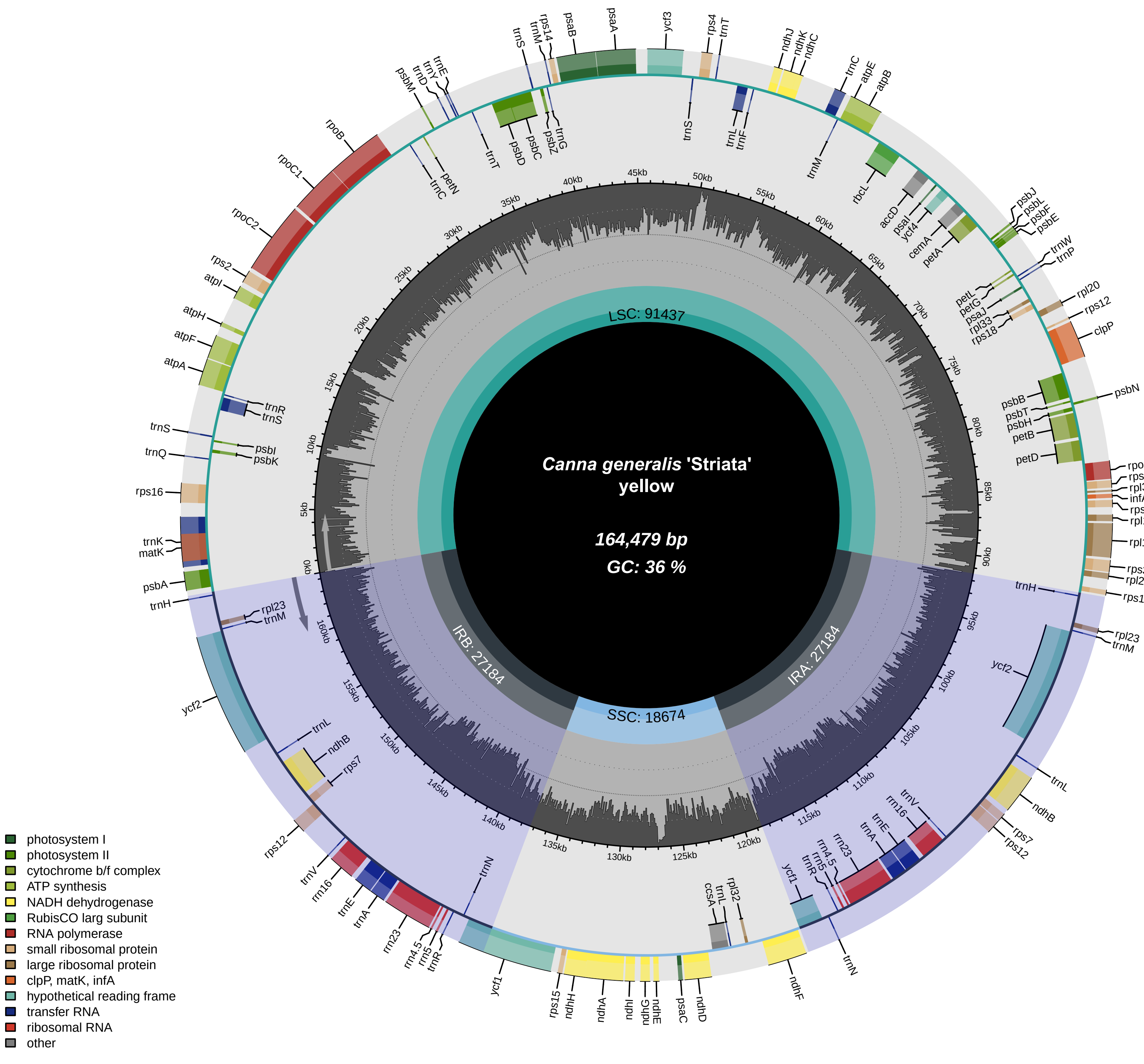
Canna generalis 'Striata'
yellow
164,479 bp
GC: 36 %
LSC: 91437
IRA: 27184
SSC: 18674
IRB: 27184
photosystem I
photosystem II
cytochrome b/f complex
ATP synthesis
NADH dehydrogenase
RubisCO larg subunit
RNA polymerase
small ribosomal protein
large ribosomal protein
clpP, matK, infA
hypothetical reading frame
transfer RNA
ribosomal RNA
other
psaB
psaA
ycf3
rps4
trnT
ndhJ
ndhK
ndhC
trnC
atpE
atpB
rbcL
accD
psaI
ycf4
cemA
petA
psbJ
psbL
psbF
psbE
trnW
trnP
rpl20
rps12
clpP
psbN
psbB
psbT
psbH
petB
petD
rpoA
rps11
rpl36
infA
rps8
rpl14
rpl16
rps3
rpl22
rps19
trnH
rpl23
trnM
ycf2
trnL
ndhB
rps7
rps12
trnV
rrn16
trnE
trnA
rrn23
rrn4.5
rrn5
trnR
trnN
ycf1
ccsA
trnL
rpl32
ndhF
rps15
ndhH
ndhA
ndhI
ndhG
ndhE
psaC
ndhD
rpoB
rpoC1
rpoC2
rps2
atpI
atpH
atpF
atpA
trnR
trnS
psbI
psbK
trnQ
rps16
trnK
matK
psbA
psbM
trnD
trnY
trnE
trnS
trnM
rps14
trnT
psbD
psbC
psbZ
trnG
petN
trnC
trnS
trnL
trnF
trnM
petL
petG
psaJ
rpl33
rps18
